# Valorization of Winemaking By-Products as a Novel Source of Antibacterial Properties: New Strategies to Fight Antibiotic Resistance

**DOI:** 10.3390/molecules26082331

**Published:** 2021-04-16

**Authors:** Adriana Silva, Vanessa Silva, Gilberto Igrejas, Isabel Gaivão, Alfredo Aires, Naouel Klibi, Maria de Lurdes Enes Dapkevicius, Patrícia Valentão, Virgílio Falco, Patrícia Poeta

**Affiliations:** 1 Microbiology and Antibiotic Resistance Team (MicroART), Department of Veterinary Sciences, University of Trás-os-Montes and Alto Douro (UTAD), 5000-801 Vila Real, Portugal; adrianaa.silva95@gmail.com (A.S.); vanessasilva@utad.pt (V.S.); 2 Department of Genetics and Biotechnology, University of Trás-os-Montes and Alto Douro (UTAD), 5000-801 Vila Real, Portugal; gigrejas@utad.pt (G.I.); igaivao@utad.pt (I.G.); 3 Functional Genomics and Proteomics Unit, University of Trás-os-Montes and Alto Douro (UTAD), 5000-801 Vila Real, Portugal; 4 Associated Laboratory for Green Chemistry (LAQV-REQUIMTE), University NOVA of Lisboa, 1099-085 Lisboa, Caparica, Portugal; 5 Veterinary and Animal Research Centre (CECAV), University of Trás-os-Montes and Alto Douro (UTAD), 5001-801 Vila Real, Portugal; 6 Centre for the Research and Technology of Agro-Environmental and Biological Sciences (CITAB), University of Trás-os-Montes and Alto Douro (UTAD), 5000-801 Vila Real, Portugal; alfredoa@utad.pt; 7 Laboratory of Microorganisms and Active Biomolecules, Department of Biology, Faculty of Sciences of Tunis, University of Tunis, Tunis 1008, Tunisia; n_klibi@yahoo.fr; 8 Faculty of Agricultural and Environmental Sciences, University of the Azores, 9700-042 Angra do Heroísmo, Portugal; 9 Institute of Agricultural and Environmental Research and Technology (IITAA), University of the Azores, 9700-042 Angra do Heroísmo, Portugal; 10 Chemistry Research Centre (CQ-VR), University of Trás-os-Montes and Alto Douro (UTAD), 5000-801 Vila Real, Portugal; valentao@ff.up.pt; 11 REQUIMTE/LAQV, Laboratório de Farmacognosia, Departamento de Química, Faculdade de Farmácia, Universidade do Porto, R. Jorge Viterbo Ferreira, n° 228, 4050-313 Porto, Portugal; vfalco@utad.pt

**Keywords:** grape by-products, antibacterial activity, antibiotic resistance, phenolic compounds

## Abstract

The emergence of antibiotic-resistance in bacteria has limited the ability to treat bacterial infections, besides increasing their morbidity and mortality at the global scale. The need for alternative solutions to deal with this problem is urgent and has brought about a renewed interest in natural products as sources of potential antimicrobials. The wine industry is responsible for the production of vast amounts of waste and by-products, with associated environmental problems. These residues are rich in bioactive secondary metabolites, especially phenolic compounds. Some phenolics are bacteriostatic/bactericidal against several pathogenic bacteria and may have a synergistic action towards antibiotics, mitigating or reverting bacterial resistance to these drugs. Complex phenolic mixtures, such as those present in winemaking residues (pomace, skins, stalks, leaves, and especially seeds), are even more effective as antimicrobials and could be used in combined therapy, thereby contributing to management of the antibiotic resistance crisis. This review focuses on the potentialities of winemaking by-products, their extracts, and constituents as chemotherapeutic antibacterial agents.

## 1. Introduction

Antibiotics have revolutionized medicine and saved millions of lives since their introduction in the 20th century. Presently, however, the incidence of bacterial infections is increasing, and their treatment is often complicated by the emergence of the antibiotic resistance crisis, which has endangered the efficacy of these chemotherapeutic drugs [1].

This crisis has been attributed to widespread use and misuse of antibiotics, both in medicine and in agriculture, leading to the selection and expansion of resistant bacterial strains associated with high rates of morbidity and mortality [2,3,4]. Bacteria have developed many different mechanisms of resistance to different classes of antibiotics [5], and bacterial strains resistant to most available antibiotics are being increasingly isolated all over the world in different environmental niches, such as humans, animals [6,7], foods [8], and the environment [9], among others. With the exponential increase in antibiotic-resistant strains and their dissemination, it has become evident that the “golden era” of antibiotics is close to its end. Thus, we are entering a new era in which it is necessary and urgent to find alternatives to overcome antibiotic resistance, in order to guarantee proper treatment in case of bacterial infections [2]. Drug-resistant infections are already responsible for more than half a million deaths globally each year. The UN Interagency Coordinating Group (IACG) on Antimicrobial Resistance estimates that if the world fails to take action to control bacterial resistance and antibacterial drugs become ineffective, this societal toll will exceed 10 million each year by 2050, over 100 trillion USD will be lost in output at the global scale, and the ensuing crisis could force up to 24 million people into extreme poverty by 2030 [10].

The rapid emergence, selection, spread, and persistence of bacterial antibiotic resistances require urgency in the search for new alternatives and treatments to combat multidrug-resistant infections. Screening natural sources for bioactive compounds is still a major avenue for drug discovery; a natural origin could be attributed to 33% of the drugs introduced in the market over the period of 1981–2014 [11], and this figure rose to 69% in the period between 1981–2006 [12]. The development of novel alternative antimicrobial agents effective against resistant bacteria is difficult, time-consuming, and far from resulting in a permanent solution. However, natural products that may enhance the antimicrobial activity of commonly used antibiotics are those that have attracted most interest from the scientific community, representing a promising alternative [2,4,13,14].

For many decades, plant-derived compounds have been used to treat and prevent bacterial infections, as part of traditional healing systems. According to the United Nations Conference on Trade and Development, more than 30% of the currently available drugs are plant-derived and over 20,000 plant species have a diversity of potential uses, as food and medicine, by humans and animals [15]. However, less than 10% of these plants have been the object of pharmacological research [16]. Plants contain a wide array of antimicrobial compounds that may provide a source for novel antimicrobial drug development [17,18], which are particularly valuable because they generally do not confer resistance [11,15,19].

Grapes are one of the largest fruit crops in the world and their industrial transformation generates vast amounts of residues, wastes, sub-products, and by-products of the greatest interest—both for different industrial sectors and for the scientific community. By-products from the winemaking industry are widely available and rich in a broad range of bioactive compounds. In recent years, grape by-products have been considered as a promising alternative source for obtaining high added-value materials, due to their antioxidant [20,21] and antimicrobial activities [22,23]. Furthermore, discarding these products may cause damage to the environment. It is, therefore, necessary to develop environmentally friendlier methods for their valorization, to comply with one of the most important objectives launched by the European Union: sustainability [24,25,26].

This review highlights the use of winery by-products (grape seeds, skins, stems, and leaves) as antimicrobials, with the objective to help combat pathogens that are resistant to conventional antibiotics. This, once highly effective, life-saving class of drugs is currently ineffective for the treatment of infections caused by resistant bacteria, due to one of the biggest health problems of the 21st century: antibiotic resistance.

## 2. Residues of the Wine Industry and Their Impacts

Wine production is one of the most important agricultural activities and grapes have a very high value as a food commodity. They are one of the largest fruit crops in the world, with more than 60 million metric tons produced annually. In 2018, FAOSTAT [27] reported 79 million tons of grape production in the world, with 43.1% of production in Europe, followed by Asia (28.4%), the Americas (20%), Africa (5.8%), and Oceania (2.7%). There are about 10,000 cultivars of grapevine in the world, *Vitis vinifera* being the most widely cultivated species for wine production. It is native to southern Europe and western Asia and cultivated in all temperate regions of the world [24,28].

The winemaking process generates a considerable volume of different residues, characterized by the presence of biodegradable compounds and suspended solids. These products are prevalent during the vintage period and are considered hazardous materials if they are not properly disposed of. If discarded without any type of treatment, they may cause negative environmental and economic impacts [24,29,30]. The main environmental damages include water pollution, soil degradation, damage to vegetation, energy use, and the emission of gases and odors. Furthermore, they resist biological degradation due to their low pH and their high content of phytotoxic and antibacterial phenolic substances. Therefore, the generation of organic and solid wastes, an unavoidable consequence of winemaking, has been described as the most important environmental issue in the global wine industry [31,32,33] and is one of the pressing issues in European wine production [34]. For instance, it has been estimated that the Portuguese wine industry generates between 1.2 and 3.5 tons ha^−1^ year^−1^ of wastes or by-products during the vintage period. These residues require treatment or an adequate recovery to minimize their environmental and economic impact, and to increase their value [31,35,36].

The development of alternatives to process the amount of waste generated during certain periods has become one of the biggest challenges for wine production [37]. These by-products are classified in different types. They consist of the pruning wood, which can be used to improve the organic matter directly in the vineyard or by composting, and the main by-product of the winemaking process: grape marc or pomace, produced during the pressing of grapes, composed of grape stalks, seeds, and skins. The rest of the by-products are produced during the wine fermentation process and consist of the lees [31,35,38].

The reuse of grape pomace has been inefficient. Some of it can be fermented and used to produce marketable distilled alcoholic drinks [39]. However, it lacks essential microbial nutrients to be efficiently composted, and low digestibility limits its use as animal feed [40]. A large amount of this by-product is generated by the wine industry at high concentrations in some areas, with the associated environmental consequences of the available means for their disposal, such as incineration and landfill deposition [30].

The implementation of waste management, the development of valorization procedures, and the search for innovative solutions are challenging issues faced by this sector, but they are indispensable to the promotion of environmental sustainability in the wine industry [34,41]. The growing demand for environment-friendly modes of production, both by consumers and regulatory bodies, requires the development of more efficient winemaking processes; however, it also entails the improvement of recovery and upcycling procedures. Such procedures should aim to innovate in terms of industrial applications, and they should focus on diminishing environmental impacts, while adding value to the wine production chain [30,38,41,42,43].

## 3. Wine By-Products—Chemical Composition and Bioactive Compounds

**Figure 1:**
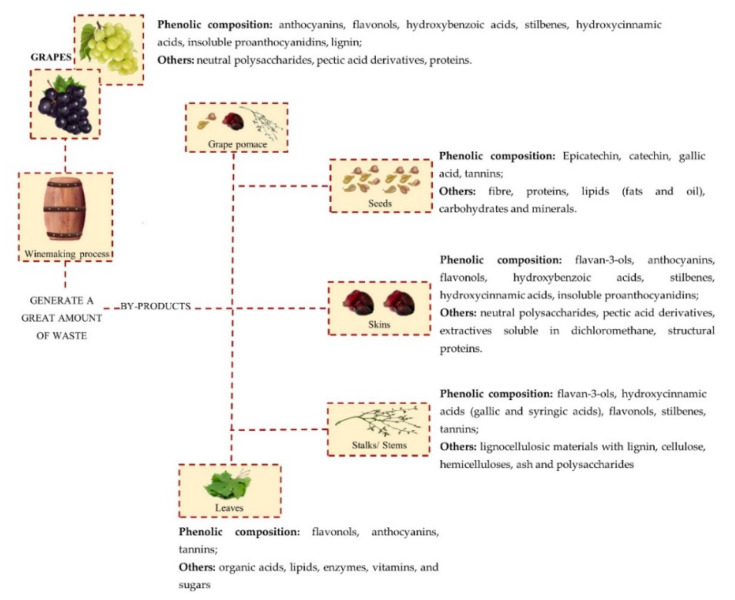
By-products generated during the winemaking process and their composition.

There is an increased interest in the valorization and use of by-products generated at the different stages of wine production, fueled by the trends of sustainable agriculture and the consumers’ preference for natural materials. As shown in Figure 1, winemaking by-products are rich in bioactive secondary plant metabolites belonging to different phytochemical groups (alkaloids, terpenes, antibiotics, volatile oils, resins, cardiac glycosides, tannins, sterols, saponins, and phenolics) [44]. They can, therefore, be valorized into a wide range of products of industrial interest, such as food additives, nutraceuticals, ingredients of foods/dietary supplements, medical remedies, fertilizers, animal feed, antimicrobial components, cosmetics, and biomass for biofuels [30,45]. Secondary metabolites are organic molecules that plants produce in response to external stressful conditions (e.g., unfavorable environment, nutrient deficiencies, pest attacks) [44], and that are not essential for their life and growth [22,46]. Rather, secondary plant metabolites (SPMs) have defensive roles and participate in interspecies competition [22]. The multiple bioactivities of SPMs make them important sources of molecules for pharmacological applications [22,47].

Polyphenols are one of the most abundant bioactive SPMs, representing ca. 70% of the bioactive compounds found in fruits [48,49]. Besides their action as plant hormones, inhibitors of enzymatic reactions, and plant growth regulators, they exhibit important properties regarding their potential for industrial use; antioxidant, antiallergenic [50], anti-inflammatory [51], anticarcinogenic [52,53], antihypertensive [54], and antibacterial [55] activities have been described in this class of compounds. Phenolic compounds are formed by an aromatic ring bearing one or more hydroxyl substituents and may range from simple phenolic molecules to highly polymerized molecules. They can be divided into two main classes on the basis of their chemical structure, number of aromatic rings, and their binding affinity for different compounds [49]: flavonoids and non-flavonoids. Flavonoids, the most abundant polyphenolics in grapes [56], can be further divided into five subclasses: anthocyanidins, flavonols, flavan-3-ols, flavones, and chalcones, depending on the degree of oxidation of the central pyran ring. Non-flavonoids include phenolic acids (hydroxybenzoic acids and hydroxycinnamic acids), stilbenes, coumarins, and lignans (Table 1) [2,24,57].

The main by-product of the winemaking industry is grape marc (or grape pomace), that is, the solid organic material that remains from the crushing, draining, and pressing processes. It consists of a mixture of grape stalks/stems, skins, and seeds. It is considered to be the most abundant winemaking by-product—it is estimated that 6 L of wine generates 1 kg of grape pomace, corresponding to 20–30% of the original grape weight [25,29,30,45]. Vast amounts of grape mark are, therefore, available in wine-producing regions of the world and disposing of them constitutes an important environmental issue. Little thought is, however, given to its potential as a source of commercially valuable products [45,58]. The chemical composition of marc can be affected by environmental (climate, soil type) [25,29,59] and viticultural factors (fertilization, defoliation, grape variety, maturity, and harvest time) [59,60], as well as by grape processing techniques employed during winemaking [59]. Whereas red wine undergoes a fermentation process, the musts are removed before alcoholic fermentation in the other wine varieties. Therefore, the fermentable sugars remain in their pomaces [29]. The composition of white grape extracts, in general, is not considerably distinct from that of the extracts of red grape cultivars, except for lower concentrations of polyphenols, particularly, a low or non-detectable amount of anthocyanins (the compounds that are responsible for the red pigmentation in grapes) [61]. Thus, the compositional differences reported by Venkitasamy et al. [29] are possibly due to the difference in the winemaking processes used. Overall, the composition of marc is complex; it contains 30% neutral polysaccharides, 20% pectic acid derivatives, and 15% insoluble proanthocyanidins, lignin, proteins, and phenols [25,29,30,62,63]. The high polyphenol content is one of the most important characteristics of grape marc, due to the pharmacological properties of this fraction, which includes resveratrol, procyanidins/condensed tannins, anthocyanins, flavanols, flavonol glycosides, phenolic acids, alcohols, lignans, stilbenes, and flavanols/catechins. Protocatechuic acid is the most dominant phenolic acid in grape pomace and the main flavonol is quercetin-3-O-glucuronide [29,45,64]. Each of the main components of grape marc—skins, seeds, and stalks/stems—has a characteristic constitution, with some presenting a more favorable composition than others, regarding their bioactive potentialities [45,58].

Grape skins are generally regarded as one of the major components of the grape pomace [45,62]. In red pomace, they correspond to 52–56% of dry matter and in white pomace to 17–28% of dry matter [62]. Phenolics are located in the inner layer of the skin, which contains the largest amount of anthocyanins and tannins with a higher polymerization degree, but a lower amount of gallates when compared with other grape pomace components [65]. The major phenols present in grape skins are flavan-3-ols, anthocyanins, flavonols, hydroxybenzoic acids (protocatechuic and gallic acid are the most dominant), stilbenes, and hydroxycinnamic acids. Anthocyanins are mainly found in skins, the most abundant being malvidin-3-O-glucoside, followed by peonidin-3-O-glucoside. The skins also present neutral polysaccharides (20% cellulose and 12% hemicelluloses), 20% of acidic pectin substances, 15% insoluble proanthocyanidins, 2–8% of ash, 5% of extractives soluble in dichloromethane, and 5–12 % structural proteins [62,64,66,67].

Grape seeds are another of the major industrial winemaking by-products; they represent 38–52% of grape pomace on a dry matter basis and about 5% of the grape weight [68]. Each year, the winemaking industry discards, worldwide, an estimate 3 Mton of grape seeds [62]. Their chemical composition depends on a variety of factors, such as climate, soil, grape variety, and degree of ripeness. Grape seeds have a high content of fiber (40% *w*/*w*), proteins (11% *w*/*w*), lipids (fats and oil; 16% *w*/*w*), polyphenolic compounds (7% *w*/*w*, such as tannins), carbohydrates, and minerals [40,69]. Grape seeds extracts are rich sources of linoleic acid and bioactive polyphenols, such as resveratrol and oligomeric procyanidins. Epicatechin, catechin, and gallic acid are the major polyphenols in grape seeds. Regarding the non-flavonoids, phenolic acids (caffeic, gallic, protocatechuic, 4-hydroxybenzoic, and syringic) are the most prevalent [29,62,70,71].

Grape stalks are the framework of the grape raceme that is removed before the vinification process. They represent 14% in weight of the total wine solid wastes and 3–5% of the raw matter of the processed grape [67]. The chemical composition of the stalks is characterized by the presence of lignocellulosic materials, with 17–26% lignin, 20–30% cellulose, 3–20% hemicelluloses, and 6–9% ash [66]. Stalks are also characterized by the presence of tannins that are associated with lignin with a higher condensation degree than other lignins from other ligneous residues [67,72]. Polysaccharides comprise more than 50% of the grape stalks. The most abundant of the extracted monosaccharides are glucose and xylose, while mannose, arabinose, and galactose have been extracted in minor quantities. As observed in the other grape by-products, the concentration of these compounds in stalks depends on several factors [62,67,72]. Grape stalks/stems contain around 6% of phenolic compounds on a dry weight basis. The main phenolic compounds are flavan-3-ols, hydroxybenzoic acids (gallic and syringic acids), flavonols (quercetin derivatives, such as glucuronide, glucoside, galactoside, and rutinoside), and stilbenes. Tannins represent approximately 80% of the phenolic compounds in grape stalks [64,67,73].

Grape leaves have a diverse chemical composition and high content in phenolic compounds. With regard to their chemical composition, they contain organic acids, flavonols, tannins, anthocyanins, lipids, enzymes, vitamins, and sugars [64].

## 4. Wine By-Products as a Source of Compounds Possessing Antibacterial Properties

Natural products are still an important source of alternative antimicrobials [16,74,75,76]. In particular, plant polyphenols may offer promise in this respect [2,24,77,78]. Besides their well-documented antibacterial properties [71,79], subinhibitory concentrations of polyphenols—either in their purified form or as polyphenol-rich plant extracts—can also enhance the activity of antibiotics when used in conjunction with these drugs, rendering formerly resistant bacteria sensitive and decreasing the antibiotic dose needed to kill or inhibit a pathogen [74].

The mechanisms underlying the antibacterial activity of polyphenols are not yet fully understood. Polyphenols are thought to target several bacterial cell constituents (cell wall, cell membrane, bacterial proteins, bacterial adhesion structures), interfere with bacterial metabolite and ion equilibria, impair the proton gradient required for oxidative phosphorylation, inhibit biofilm formation, and interfere with nucleic acid synthesis and with the regulation of gene expression [2,18,24,80]. Furthermore, they may attenuate virulence [81]. The bacterial cell wall seems to be the main target for the antibacterial action of phenolic compounds [82]. They compromise cell wall integrity, leading to increased permeability and deformation of the cell [80]. The outer membrane present in the cell wall of Gram-negative bacteria may hinder polyphenol uptake; therefore, Gram-positive bacteria may appear more sensitive than the Gram-negative bacteria [77,83]. The cell membrane is another important target for some polyphenols (e.g., ellagitannins, catechins, non-lignans). Some have high affinity for bacterial membranes, particularly for those of Gram-positive bacteria, affecting membrane thickness and fluidity, and increasing its permeability [18,80,84,85]. Polyphenols are also capable of bonding covalently and non-covalently (hydrogen bonds, hydrophobic interactions, van der Waals attractions) with important bacterial proteins, such as penicillin-binding proteins (PBPs), transporter proteins, surface-adhesion proteins, membrane-bound enzymes, and cell-wall polypeptides. The interaction polyphenols–PBPs is of special importance for their potentialities as antibiotic adjuvants [80]. Certain polyphenols can modulate bacterial gene expression, thereby causing major metabolic changes. The exact mechanisms of this modulation are not yet known; it may be achieved either by direct interaction with the bacterial DNA or by epigenetic mechanisms (modulation of the activity of transcription factors [80]. Additionally, polyphenols inhibit several enzymes involved in nucleic acid synthesis (topoisomerases, gyrases, and helicases), a property that significantly contributes to their antimicrobial activity [18]. Polyphenols can modulate the concentrations of essential bacterial metabolites, impair ionic strength equilibria, and weaken the proton gradient, thereby killing the target bacteria [85]. Bacterial biofilms allow bacteria to persist in the environment and take part in their pathogenic activity [86]. Polyphenols have been proven effective against bacterial biofilms [80,87]. These compounds influence biofilm establishment and maturation by interfering with bacterial adhesion, motility, and quorum sensing [18]. One potential application of polyphenols with antibiofilm properties is in the treatment of urinary tract infections [80]. They may also prove useful in the prevention of biofilms on the surface of medical devices [87]. Phenolic compounds can act as antioxidants due to the presence of free -OH groups, which inhibit the generation of reactive oxygen species and scavenge free radicals, thus decreasing the redox potential and affecting microbial growth [13].

The antibacterial activity of polyphenols depends on their chemical structure and it seems to be related with the presence of the 3,4,5-trihydroxifenyl group [24]. Polyphenols are a diverse class of molecules, with differences in the structure of their carbon framework, presence of glycosidic bonds, and number, position, and alkylation of OH groups [82,88,89,90,91,92] that account for the differences in their antibacterial activity. The antibacterial spectrum of polyphenols, as a class of molecules, has been reported to encompass both Gram-negative and Gram-positive bacteria [79], albeit the former have been touted as less sensitive to their action [83]. Antibacterial activity against antibiotic-resistant and non-antibiotic resistant Gram-positive bacteria has been demonstrated in several polyphenols (phenolic acids, flavonoids, tannins, lignans, and stilbenes) and their combinations [80]. The sensitivity towards individual phenolic compounds is not only species but also strain dependent [83]. Mixtures of polyphenols, such as those present in plant extracts, appear to have a stronger inhibitory activity than individual compounds [77].

Several studies have demonstrated the antibacterial potential of extracts from winemaking by-products (WBPEs) [92,93,94,95,96,97,98,99,100,101,102,103,104,105,106,107,108]. The phenolics that are responsible for the antimicrobial activity in winemaking by-products are phenolic acids, quinones, saponins, flavonoids, tannins, coumarins, terpenoids, and alkaloids [2,13,24]. However, there is a wide structural variation among the major bioactive compounds, and this can result in differences in their antimicrobial action, as discussed above.

Table 2 presents an overview of the studies on the antibacterial activity of WBPEs. The paucity of studies concerning the antimicrobial activities of WBPEs and the diversity of methodologies they employ complicate attempts to derive broad conclusions from their analysis. Inhibitory activity against a broad range of Gram-positive (*Bacillus amyloliquefaciens*, *Bacillus brevis*, *Bacillus cereus*, *Bacillus coagulans*, *Bacillus subtilis*, *Clostridium septicum*, *Enterococcus faecalis*, *Enterococcus faecium*, *Listeria monocytogenes*, *Mycobacterium smegmatis*, *Staphylococcus aureus*, and *Staphylococcus epidermidis*) and Gram-negative bacteria (*Aeromonas hydrophila*, *Campylobacter coli*, *Enterobacter aerogenes*, *Escherichia coli*, *Klebsiella pneumoniae*, *Proteus vulgaris*, *Pseudomonas aeruginosa*, *Shigella sonnei*, as well as the Enteritidis, Infantis, Poona, and Typhimurium serovars of *Salmonella enterica*) [92,93,94,95,96,97,98,99,100,101,102,103,104,105,106]. Interestingly, WBPEs were active against multi-resistant strains in some cases [99,104] and activity was also observed against bacteria that have passive mechanisms of antibiotic exclusion, like the spore protective layers in sporulated genera (*Bacillus*, *Clostridium*) [94,95,97,98,99,102,103,104,105,106] or the cell wall of acid-fast bacteria (*Mycobacterium*) [97,108]. In some studies, a broader inhibitory spectrum and/or lower minimum inhibitory concentrations (MICs) were found against Gram-positive bacteria rather than against Gram negatives [107]. Frequently, WBPEs (e.g., seed extracts) are bactericidal [108]. The minimum inhibitory concentrations (MICs) presented in Table 2. vary widely, reflecting not only the resistance/sensitivity of the target bacteria, but also several factors that influence the type and concentrations of the individual phenolics present in the extracts.

When it comes to the antimicrobial activity of WBPEs, there are several factors to be considered. Extraction solvent, extraction procedures, pomace fraction, and grape variety have been found to affect the yield, polyphenolic composition, and antimicrobial activity of the extracts [96,105]. The bactericidal effect of extracts was demonstrated to be dose dependent, but often lower concentrations were also inhibitory [94,97,98,103,106]. Studies comparing the type of solvents used [94,97] have found that acetone:water:acetic acid(90:9.5:0.5) extracts had higher inhibitory activity than methanol:water:acetic acid (90:9.5:0.5) extracts. However, WBPEs obtained with other solvent systems also displayed antimicrobial activity, as shown in Table 2. Grape variety seems to affect the antibacterial activity of WBPEs in most reports [96,98,101,103]. In some [98,102], but not in all relevant studies [96,99,101], WPBEs originated from red grapes had higher MICs than those from white grapes. The studies in Table 2. show that extracts from all types of by-products (pomace, skins, seeds, stems, and leaves) displayed antibacterial activity against at least some of the bacterial species/strains under study, with some studies indicating a broader spectrum of activity for seed extracts in comparison with the extracts obtained from other winemaking by-products [94,99,109]. A link between the phenolic content of extracts obtained from the different pomace fractions and their antibacterial activity has been established in some studies [92,94,99,100,105,107]. Due to their high phenolic content, seeds appeared to have a better performance as sources of antibacterial extracts than other fractions [99]. The antibacterial activity has been related with the presence of non-flavonoids (gallic acid [92,100,105], ethyl gallate, caffeic acid, tyrosol, tryptophol [94], p-OH-benzoic acid, vanillic acid [105], and, less often, to flavonoids (catechin, epicatechin) and stilbenes (trans-resveratrol) [99] in the WBPEs. Phenolic extracts showed, in general, higher antimicrobial activity when compared to pure phenolic compounds, indicating a possible synergistic effect between the constituents of the complex mixtures that constitute the extracts [92]. A synergic effect is apparent not only when polyphenols are part of complex mixtures, but also when they are present, simultaneously, with an antibiotic. In this situation, subinhibitory concentrations of plant polyphenols may restore antibiotic susceptibility in bacteria that had previously gained resistance [74,110].

## 5. Wine By-Products in the Combat against Antibiotic Resistance

Plant extracts (and their antimicrobial constituents) may be of use in the constant fight of humankind against bacterial pathogens in two main ways: (i) as novel antimicrobials, because they inhibit pathogen growth and/or threaten their viability, or (ii) as therapeutic adjuvants, because they can modulate bacterial virulence and/or act as antibiotic resistance modifiers [110].

The relatively broad spectrum of activity and the promising results of in vitro assessment of their antibacterial activity makes some extracts from plant materials, such as winemaking by-products, an attractive research avenue for the development of novel antimicrobials. As with other plant-derived materials, passage through the gastrointestinal tract may result in degradation [111], although encapsulation may offer a solution to avoid loss of activity and ensure an adequate drug delivery [112]. Furthermore, in other body sites, the presence of mucus or plasma proteins may compromise in vivo efficacy. Besides their potential applications in the management of urinary tract infections and for the prevention of biofilm formation on medical devices [80,87], topical application is more practicable and could help reduce antibiotic use in certain types of infections [84].

The main possible application that polyphenol-rich extracts from winemaking by-products may find as antimicrobials is, possibly, in combination therapy, as antibiotic adjuvants [84,110,113]. Combination therapy may provide a means to circumvent bacterial resistance to antibiotics, by avoiding monotherapy, expanding the spectrum, enhancing their bactericidal/bacteriostatic activity, and preventing the emergence of antibiotic-resistant mutants. This could revert the course of infections that do not respond to conventional antibacterial chemotherapy, including those that are caused by multi-resistant strains [113]. The potential of WBPEs as multidrug resistance inhibitors, thereby enhancing the activity of the existing antibiotics, is one of the most promising aspects of their multiple bioactivities [74,80,110,113].

Knowledge on the mechanisms of plant polyphenols–antibiotic synergism is still incomplete. Four main processes seem to be involved [110]:

(i). Modification of the active sites in and on the bacterial cell, e.g., PBPs, topoisomerases [80];
(ii). Inhibition of bacterial enzymes involved in antibiotic modification or degradation, e.g., β-lactamase inhibition [114];
(iii). Increased membrane permeability [80]; and
(iv). Inhibition of antibiotic efflux pumps, such as Tet(K) in *S. aureus* and *S. epidermidis* [80].

Synergism between grape pomace extracts and antibiotics belonging to different classes (fluoroquinolones, β-lactams, amphenicols, and tetracyclines) has been demonstrated in vitro against multidrug-resistant clinical strains of *E. coli* and *S. aureus* [115]. The discovery of in vitro bioactive properties is, however, the very beginning of the long winding road towards drug development. Bioavailability, toxicity, mode of delivery, and interaction with other components or drugs in a clinical setting are equally important [23,84,116]. In addition to these, the present scarcity of knowledge on the synergetic properties of WBPEs as well as on their mechanisms of interaction with bacteria and with the antibiotics constitute important bottlenecks and make further research a necessity. Another obstacle that impedes a clear view on the potentialities of WBPEs as antimicrobials and/or antibiotic adjuvants is the lack of standardization in the analytical methodologies used to evaluate antimicrobial activity and synergism in the different studies [12,22,23,81,84].

## 6. Methods

A literature search was performed in the Web of Science and in PubMed. Google Scholar was used to identify relevant grey literature. The search terms used were “antibiotic resistance”, “winemaking by-products”, “grape by-products”, “natural products”, and “antibacterial properties”. No restrictions about publication type or year were applied. The search was conducted in April 2020. The winemaking by-products that were considered were pomace, leaf, skins, seeds, and stalks/stems. The development and emergence of multidrug-resistant bacteria has increased over the past years and the reduction of new antibacterial drugs in the pharmaceutical industry has begun to decline. This review summarized studies on the antimicrobial activity of extracts from grape by-products and demonstrates that these extracts are promising, safe, and cheap for the development of novel therapies, especially aimed at infections caused by Gram-positive bacteria. Further studies should focus on in vivo tests to define the usefulness of these antibacterial agents in medicine.

## 7. Conclusions

Due to their antimicrobial activity and synergism with antibiotics, WBPEs may be part of the arsenal required to control antibiotic resistance while responding to another pressing social issue: the need to ensure the environmental sustainability of agricultural production. There are gaps in the presently existing knowledge that demand a considerable research effort and, as is the case with all areas of natural product research, the road from lab to product is long and can be challenging. However, the high content of these by-products (especially grape seeds) in a well-documented class of bioactive compounds—polyphenols—makes them a promising avenue for the search of novel classes of antibacterial drug enhancers and adds value to a type of residue that is difficult to dispose of.

## Figures and Tables

**Table 1:** Main classes of phenolic compounds.

Phenolic Compounds		
Flavonoids	Non-Flavonoids	
Anthocyanidins 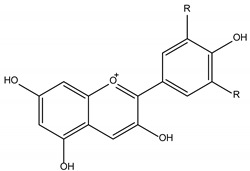	Phenolic Acids	
	Hydroxybenzoic acids derivates 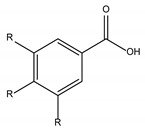	Hydroxycinnamic acids derivates 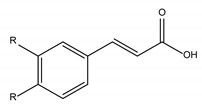
Flavonols 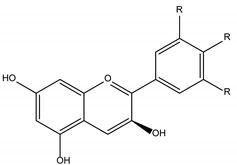	Stilbenes 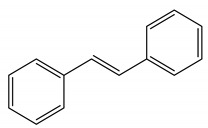	
Flavan-3-ols 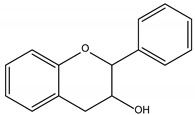	Coumarins 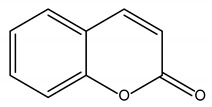	
Flavones 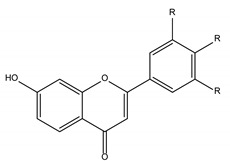	Lignans 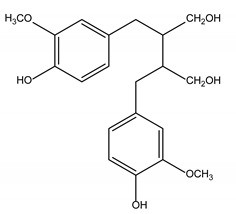	
Chalcones 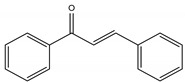		

**Table 2:** Grape by-products as antimicrobials.

Grape Variety	Country	By-Product	Extraction Solvent(s)/Methodologies	Target Bacteria		MIC Range (mg mL^−1^)	Reference
				Gram-Positive	Gram-Negative		
Arinto	Portugal	Skins/Seeds	Water	*B. cereus*	*E. coli* *S.* Poona	ND	[103]
Touriga Nacional	Portugal	Skins	Water:ethanol (50:50)	*B. cereus* *E. faecium* *L. monocytogenes* *S. epidermidis*	*K. pneumoniae*	0.01–1.0	[99]
		Stems		*E. faecium* *L. monocytogenes* *S. aureus* *S. epidermidis*	-	0.05–0.1	
		Seeds		*B. cereus* *E. faecalis* *E. faecium* *L. monocytogenes* *S. epidermidis* *S. aureus*	*K. pneumoniae*	0.01–0.1	
Preto Martinho		Skins		*E. faecalis* *L. monocytogenes* *S. epidermidis* *S. aureus*	-	0.01–0.075	
		Stems		*E. faecalis* *E. faecium* *L monocytogenes* *S. epidermidis*	*K. pneumoniae*	0.025–0.1	
		Seeds		*B. cereus* *E. faecalis* *L. monocytogenes* *S. epidermidis* *S. aureus*	*K. pneumoniae*	0.001–0.01	
Noble	USA	Skins	Methanol:water (70:30)	*S. aureus* (ATCC 35548) *S. aureus* (ATCC 12600) *S. aureus* (ATCC 29247)	*S. sonnei*	226–903	[100]
		Seeds				268–1069	
Carlos		Skins				304–608	
		Seeds				268–1069	
**Grape Variety**	**Country**	**By-Product**	**Extraction Solvent(s)/Methodologies**	**Gram-Positive**	**Gram-Negative**	**MIC Range (mg mL^−1^)**	**Reference**
Kujundžuša	Croatia	Skins	Ethanol:water (80:20)	*B. cereus* *S. aureus*	*C. coli* *E. coli* *S.* Infantis	0.032–0.15 *	[102]
Rkaciteli						0.014–0.20 *	
Zlatarica						0.042–0.59 *	
Medna						0.014–0.21 *	
Kuč						0.019–0.26 *	
Maraština						0.015–0.21 *	
Debit						0.025–0.25 *	
Vranac						0.16–0.23 *	
Trnjac						0.12–0.31 *	
Rudežuša						0.15–0.29 *	
Merlot						0.13–0.44 *	
Babić						0.08–0.42 *	
Lain						0.04–0.34 *	
Plavina						0.09–0.41 *	
Autumn Royal	India	Leaf	Methanol	*C. septicum* *S. aureus* (MRSA) *S. viridans*	*E. coli* *Proteus* spp. *Pseudomonas* spp.	ND	[104]
Crimson							
Thompson							
Sundarkhani							
Perlette							
King’s Ruby							
Viognier	USA	Pomace	Acetone:water (80:20)	*L. monocytogenes* *S. aureus*	-	5.07–40.6	[100]
Vidal Blanc						15.6–250	
Cabernet Franc						4.69–75	
Chambourcin						18.8–75	
Bangalore blue	India	Seeds	Acetone:water:acetic acid (90:9.5:0.5)	*B. cereus* *B. coagulans* *B. subtilis* *S. aureus*	*E. coli* *P. aeruginosa*	ND	[97]
			Methanol:water:acetic acid (90:9.5:0.5)				
Palavá	Slovakia	Pomace	ethanol	*B. ceresu* *S. aureus*	*E. coli* *P. aeruginosa*	0.5–2	[98]
Dornfelder							
Narince	Turkey	Bagasse	Ethyl acetate:methanol:water (60:30:10)	-	-	ND	[94]
			Ethanol:water (95:5)				
**Grape Variety**	**Country**	**By-Product**	**Extraction Solvent(s)/Methodologies**	**Gram-Positive**	**Gram-Negative**	**MIC Range (mg mL^−1^)**	**Reference**
Narince	Turkey	Defatted seeds	Acetone:water:acetic acid (90:9.5:0.5)	*B. amyloliquefaciens* *B. brevis*	*P. vulgaris* *P. aeruginosa*	ND	[94]
			Ethyl acetate:methanol:water (60:30:10)				
Hasandede	Turkey	Defatted seeds	Acetone:water:acetic acid (90:9.5:0.5)	*B. cereus* *E. faecalis* *M. smegmatis* *S. aureus*	*A. hydrophyla* *E. aerogenes* *E. coli* *K. pneumoniae* *P. vulgaris* *P. aeruginosa* *S.* Enteritidis *S.* Typhimurium	ND	[95]
Emir							
Kalecic Karasi							
Pinot Noir	New Zealand	Pomace	Acetone:water (50:50)	*S. aureus*	*E. coli*	0.39–25	[96]
			Ethanol:water (50:50)			0.78–25	
			Methanol:water (50:50)			0.78–25	
		Seeds	Acetone:water (50:50)			0.39–25	
			Ethanol:water (50:50)			0.78–25	
			Methanol:water (50:50)			0.195–25	
		Skins	Acetone:water (50:50)			0.39–25	
			Ethanol:water (50:50)			0.78–25	
			Methanol:water (50:50)			12.5–25	
**Grape Variety**	**Country**	**By-Product**	**Extraction Solvent(s)/Methodologies**	**Gram-Positive**	**Gram-Negative**	**MIC Range (mg mL^−1^)**	**Reference**
Pinot Meunier	New Zealand	Pomace	Acetone:water (50:50)	*S. aureus*	*E. coli*	12.5–25	[96]
			Ethanol:water (50:50)			12.5	
			Methanol:water (50:50)			12.5	
		Seeds	Acetone:water (50:50)			3.125–25	
			Ethanol:water (50:50)			1.56–100	
			Methanol:water (50:50)			0.195–25	
		Skins	Acetone:water (50:50)			12.5–25	
			Ethanol:water (50:50)			12.5–25	
			Methanol:water (50:50)			25	
Merlot	Brazil	Pomace	SFE-ethanol	*B. cereus* *S. aureus*	*E. coli* *P. aeruginosa*	0.007–0.012	[105]
			SOX-hexane			-	
Syrah	Brazil	Pomace	SFE-ethanol	*B. cereus* *S. aureus*	-	-	
			SOX-hexane	*B. cereus*		0.014	

* MIC values are in GAE mL^−1^; SFE–Supercritical Fluid Extraction; SOX–Sohxlet Extraction.

## Data Availability

Not applicable.

## References

[1] Abadi T.B., Rizvanov A.A., Haertlé T., Blatt N.L. (2019). World Health Organization Report: Current crisis of antibiotic resistance. BioNanoScience.

[2] Miklasińska-Majdanik M., Kępa M., Wojtyczka R.D., Idzik D., Wąsik T.J. (2018). Phenolic compounds diminish antibiotic resistance of *Staphylococcus aureus* clinical strains. Int. J. Environ. Res. Public Health.

[3] Ventola C.L. (2015). The antibiotic resistance crisis: Part 1: Causes and threats. Pharm. Ther..

[4] Li B., Webster T.J. (2018). Bacteria antibiotic resistance: New challenges and opportunities for implant-associated orthopedic infections. J. Orthop. Res..

[5] Hobson C., Chan A.N., Wright G.D. (2021). The antibiotic resistome: A guide for the discovery of natural products as antimicrobial agents. Chem. Rev..

[6] Ramey A.M., Ahlstrom C.A. (2020). Antibiotic resistant bacteria in wildlife: Perspectives on trends, acquisition and dissemination, data gaps, and future directions. J. Wildl. Dis..

[7] Palma E., Tilocca B., Roncada P. (2020). Antimicrobial resistance in veterinary medicine: An overview. Int. J. Mol. Sci..

[8] Caniça M., Manageiro V., Abriouel H., Moran-Gilad J., Franz C.M.A.P. (2019). Antibiotic resistance in foodborne bacteria. Trends Food Sci. Technol..

[9] Moussally M., Zahreddine N., Kazma J., Ahmadieh R., Kan S.S., Kanafan Z.A. (2021). Prevalence of antibiotic-resistant organisms among hospitalized patients at a tertiary care center in Lebanon, 2010–2018. J. Infect. Public Health.

[10] IACG/WHO (2019). No time to wait: Securing the future from drug-resistant infections. Report to the Secretary-General of the United Nations of the Interagency Coordination Group on Antimicrobial Resistance.

[11] Newman D.J., Cragg G.M. (2016). Natural products as sources of new drugs from 1981 to 2014. J. Nat. Prod..

[12] Savoia D. (2012). Plant-derived antimicrobial compounds: Alternatives to antibiotics. Future Microbiol..

[13] Gyawali R., Ibrahim S.A. (2014). Natural products as antimicrobial agents. Food Control.

[14] WHO (2001). Global Strategy for Containment of Antimicrobial Resistance.

[15] Cheesman M.J., Ilanko A., Blonk B., Cock I.E. (2018). Developing new antimicrobial therapies: Are synergistic combinations of plant extracts/compounds with conventional antibiotics the solution?. Pharm. Rev..

[16] Atanasov A.G., Waltenberger B., Pferschy-Wenzig E.M., Linder T., Wawrosch C., Uhrin P., Temml V., Wang L., Schwaiger S., Heiss E.H. (2015). Discovery and resupply of pharmacologically active plant-derived natural products: A review. Biotechnol. Adv..

[17] Anand U., Jacobo-Herrera N., Altemimi A., Lakhssassi N. (2019). A comprehensive review on medicinal plants as antimicrobial therapeutics: Potential avenues of biocompatible drug discovery. Metabolites.

[18] Górniak I., Bartoszewski R., Króliczewski J. (2019). Comprehensive review of antimicrobial activities of plant flavonoids. Phytochem. Rev..

[19] Mahady G. (2005). Medicinal Plants for the Prevention and Treatment of Bacterial Infections. Curr. Pharm. Des..

[20] Tungmunnithum D., Thongboonyou A., Pholboon A., Yangsabai A. (2018). Flavonoids and other phenolic compounds from medicinal plants for pharmaceutical and medical aspects: An overview. Medicines.

[21] Krishnaiah D., Sarbatly R., Nithyanadam R. (2011). A review of the antioxidant potential of medicinal plant species. Food Bioprod. Process..

[22] Barbieri R., Coppo E., Marchese A., Daglia M., Sobarzo-Sánchez E., Nabavi S.F., Nabavi S.M. (2017). Phytochemicals for human disease: An update on plant-derived compounds antibacterial activity. Microbiol. Res..

[23] Mattos G.N., Tonon R.V., Furtado A.A.L., Cabral L.M.C. (2017). Grape by-product extracts against microbial proliferation and lipid oxidation: A review. J. Sci. Food Agric..

[24] Brenes A., Viveros A., Chamorro S., Arija I. (2016). Use of polyphenol-rich grape by-products in monogastric nutrition. A review. Anim. Feed Sci. Technol..

[25] Devesa-rey R., Vecino X., Varela-alende J.L., Barral M.T., Cruz J.M., Moldes A.B. (2011). Valorization of winery waste vs. the costs of not recycling. Waste Manag..

[26] (2018). FAOSTAT, Food and Agriculture Organization of the United Nations—Statistics Division. http://www.fao.org/faostat/.

[27] Barba F.J., Zhu Z., Koubaa M., Sant’Ana A.S., Orlien V. (2016). Green alternative methods for the extraction of antioxidant bioactive compounds from winery wastes and by-products: A review. Trends Food Sci. Technol..

[28] Venkitasamy C., Zhao L., Zhang R., Pan Z., Pan Z., Zhang R., Zicaria S. (2019). Chapter 6. Grapes. Integrated Processing Technologies for Food and Agricultural By-Products.

[29] Kalli E., Lappa I., Bouchagier P., Tarantilis P.A., Skotti E. (2018). Novel application and industrial exploitation of winery by-products. Bioresour. Bioprocess..

[30] Oliveira M., Duarte E. (2016). Integrated approach to winery waste: Waste generation and data consolidation. Front. Environ. Sci. Eng..

[31] Christ K.L., Roger L. (2013). Burritt. Critical environmental concerns in wine production: An integrative review. J. Clean. Prod..

[32] Bustamante M.A., Moral R., Paredes C., Pérez-Espinosa A., Moreno-Caselles J., Pérez-Murcia M.D. (2008). Agrochemical characterisation of the solid by-products and residues from the winery and distillery industry. Waste Manag..

[33] Mendes J.A.S., Prozil S.O., Evtuguin D.V., Lopes L.P.C. (2013). Towards comprehensive utilization of winemaking residues: Characterization of grape skins from red grape pomaces of variety Touriga Nacional. Ind. Crop. Prod..

[34] Nogales R., Cifuentes C., Benítez E. (2005). Vermicomposting of winery wastes: A laboratory study. J. Environ. Sci. Health Part B Pestic. Food Contam. Agric. Wastes.

[35] Brito P.S.D., Oliveira A.S., Rodrigues L.F. (2014). Energy valorization of solid vines pruning by thermal gasification in a pilot plant. Waste Biomass Valorization.

[36] Sánchez-Gómez R., Alonso G.L., Salinas M.R., Zalacain A. (2017). Reuse of Vine-Shoots Wastes for Agricultural Purposes.

[37] Ruggieri L., Cadena E., Martínez-Blanco J., Gasol C.M., Rieradevall J., Gabarrell X., Gea T., Sort X., Sánchez A. (2009). Recovery of organic wastes in the Spanish wine industry. Technical, economic and environmental analyses of the composting process. J. Clean. Prod..

[38] Kokkinomagoulos E., Kandylis P. (2020). Sustainable exploitation of by-Products of vitivinicultural origin in winemaking. Proceedings.

[39] Antonić B., Jančíková S., Dordević D., Tremlová B. (2020). Grape pomace valorization: A systematic review and meta-analysis. Foods.

[40] Troilo M., Difonzo G., Paradiso V.M., Summo C., Caponio F. (2021). Bioactive compounds from vine shoots, grape stalks, and wine lees: Their potential use in agro-food chains. Foods.

[41] Mateo J.J., Maicas S. (2015). Valorization of winery and oil mill wastes by microbial technologies. Food Res. Int..

[42] Gómez-Brandón M., Lores M., Insam H., Domínguez J. (2019). Strategies for recycling and valorization of grape marc. Crit. Rev. Biotechnol..

[43] Ali K., Maltese F., Choi Y.H., Verpoorte R. (2010). Metabolic constituents of grapevine and grape-derived products. Phytochem. Rev..

[44] Graça A., Corbet-Milward J., Schultz H.R., Ozer C., de la Fuente M. (2018). Managing By-Products of Vitivinicultural Origin.

[45] Lelario F., Scrano L., De Franchi S., Bonomo M.G., Salzano G., Milan S., Bufo S.A. (2018). Identification and antimicrobial activity of most representative secondary metabolites from different plant species. Chem. Biol. Technol. Agric..

[46] Gorlenko C.L., Kiselev H.Y., Budanova E.V., Zamyatnin A.A., Ikryannikova L.N. (2020). Plant secondary metabolites in the battle of drugs and drug-resistant bacteria: New heroes or worse clones of antibiotics?. Antibiotics.

[47] Abegaz B.M., Kinfe H.H. (2019). Secondary metabolites, their structural diversity, bioactivity, and ecological functions: An overview. Phys. Sci. Rev..

[48] Swallah M.S., Sun H., Affoh R., Fu H., Yu H. (2020). Antioxidant potential overviews of secondary metabolites (polyphenols) in fruits. Int. J. Food Sci..

[49] Lipiński K., Mazur M., Antoszkiewicz Z., Purwin C. (2017). Polyphenols in monogastric nutrition—A review. Ann. Anim. Sci..

[50] Shahidi F., Ambigaipalan P. (2015). Phenolics and polyphenolics in foods, beverages and spices: Antioxidant activity and health effects—A review. J. Funct. Foods.

[51] Ambriz-Pérez D.L., Leyva-López N., Erick P., Gutierrez-Grijalva E.P., Heredia J.B. (2016). Phenolic compounds: Natural alternative in inflammation treatment. A Review. Cogent Food Agric..

[52] Roleira F.M., Tavares-da-Silva E.J., Varela C.L., Costa S.C., Silva T., Garrido J., Borges F. (2015). Plant derived and dietary phenolic antioxidants: Anticancer properties. Food Chem..

[53] Salehi B., Vlaisavljevic S., Adetunji C.O., Adetunji J.B., Kregiel D., Antolaki H., Pawlikowska E., Uprety Y., Mileski K.S., Devkota H.P. (2019). Plants of the genus *Vitis*: Phenolic compounds, anticancer properties and clinical relevance. Trends Food Sci. Technol..

[54] Al Shukor N., Van Camp J., Gonzalez G.B., Staljanssens D., Struijs K., Zotti M.J., Raes K., Smagghe G. (2013). Angiotensin-converting enzyme inhibitory effects by plant phenolic compounds: A study of structure activity relationships. J. Agric. Food Chem..

[55] Rempe C.S., Burris K.P., Lenaghan S.C., Stewart C.N. (2017). The potential of systems biology to discover antibacterial mechanisms of plant phenolics. Front. Microbiol..

[56] Averilla J.N., Oh J., Kim H.J., Kim J.S., Kim J.-S. (2019). Potential health benefits of phenolic compounds in grape processing by-products. Food Sci. Biotechnol..

[57] Vinholes J., Silva B.M., Silva L.R., Berhardt L.V. (2010). Hydroxycinnamic acids (HCAS): Structure, biological properties and health effects. Advances in Medicine and Biology.

[58] Sirohi R., Tarafdar A., Singh S., Negi T., Gaur V.K., Gnansounou E., Bharathiraja B. (2020). Green processing and biotechnological potential of grape pomace: Current trends and opportunities for sustainable biorefinery. Bioresour. Technol..

[59] Bordiga M., Travaglia F., Locatelli M. (2019). Valorisation of grape pomace: An approach that is increasingly reaching its maturity—A review. Int. J. Food Sci. Technol..

[60] Pavić V., Kujundžić T., Kopić M., Jukić V., Braun U., Schwander F., Drenjančević M. (2019). Effects of Defoliation on phenolic concentrations, antioxidant and antibacterial activity of grape skin extracts of the varieties Blaufränkisch and Merlot (*Vitis vinifera* L.). Molecules.

[61] Hassan Y.I., Kosir V., Yin X., Ross K., Diarra M.S. (2019). Grape pomace as a promising antimicrobial alternative in feed: A critical review. J. Agric. Food Chem..

[62] Dávila I., Robles E., Egüés I., Labidi J., Gullón P., Galanakis C.M. (2017). The Biorefinery Concept for the Industrial Valorization of Grape Processing By-Products. Handbook of Grape Processing By-Products. Sustainable Solutions.

[63] Deng Q., Penner M.H., Zhao Y. (2011). Chemical composition of dietary fiber and polyphenols of five different varieties of wine grape pomace skins. Food Res. Int..

[64] Hogervorst J.C., Miljić U., Puškaš V., Galanakis C.M. (2017). Extraction of Bioactive Compounds from Grape Processing By-Products. Handbook of Grape Processing By-Products. Sustainable Solutions.

[65] Muhlack R.A., Potumarthi R., Jeffery D.W. (2018). Sustainable wineries through waste valorisation: A review of grape marc utilisation for value-added products. Waste Manag..

[66] Nanni A., Parisi M., Colonna M. (2021). Wine by-products as raw materials for the production of biopolymers and of natural reinforcing fillers: A critical review. Polymers.

[67] Spigno G., Marinoni L., Garrido G.D., Galanakis C.M. (2017). State of the Art in Grape Processing By-Products. Handbook of Grape Processing By-Products. Sustainable Solutions.

[68] Beres C., Costa G.N.S., Cabezudo I., da Silva-James N.K., Teles A.S.C., Cruz A.P.G., Mellinger-Silva C., Tonon R.V., Cabral L.M.C., Freitas S.P. (2017). Towards integral utilization of grape pomace from winemaking process: A review. Waste Manag..

[69] Ma Z.F., Zhang H. (2017). Phytochemical constituents, health benefits, and industrial applications of grape seeds: A mini-review. Antioxidants.

[70] Domínguez J., Sanchez-Hernandez J.C., Lores M., Galanakis C.M. (2017). Vermicomposting of Winemaking By-Products. Handbook of Grape Processing By-Products. Sustainable Solutions.

[71] Friedman M. (2014). Antibacterial, antiviral, and antifungal properties of wines and winery byproducts in relation to their flavonoid content. J. Agric. Food Chem..

[72] Souquet J.-M., Labarbe B., Le Guernevé C., Cheynier V., Moutounet M. (2000). Phenolic composition of grape stems. J. Agric. Food Chem..

[73] Blackford M., Comby M., Zeng L., Dienes-Nagy Á., Bourdin G., Lorenzini F., Bach B. (2021). A Review on stems composition and their impact on wine quality. Molecules.

[74] Álvarez-Martínez F.J., Barrajón-Catalán E., Micol V. (2020). Tackling antibiotic resistance with compounds of natural origin: A comprehensive review. Biomedicines.

[75] Thomford N.E., Senthebane D.A., Rowe A., Munro D., Seele P., Maroyi A., Dzobo K. (2018). Natural products for drug discovery in the 21st century: Innovations for novel drug discovery. Int. J. Mol. Sci..

[76] Wright G.D. (2019). Unlocking the potential of natural products in drug discovery. Microb. Biotechnol..

[77] Efenberger-Szmechtyk M., Nowak A., Czyzowska A. (2021). Plant extracts rich in polyphenols: Antibacterial agents and natural preservatives for meat and meat products. Crit. Rev. Food Sci. Nutr..

[78] Jara-Palacios M.J., Hernanz D., Cifuentes-Gomez T., Escudero-Gilete M.L., Heredia F.J., Spencer J.P.E. (2015). Assessment of white grape pomace from winemaking as source of bioactive compounds, and its antiproliferative activity. Food Chem..

[79] Daglia M. (2012). Polyphenols as antimicrobial agents. Curr. Opin. Biotechnol..

[80] Álvarez-Martínez F.J., Barrajón-Catalán E., Encinar J.A., Rodríguez-Díaz J.C., Micol V. (2020). Antimicrobial capacity of plant polyphenols against Gram-positive bacteria: A comprehensive review. Curr. Med. Chem..

[81] AlSheikh H.M.A., Sultan I., Kumar V., Rather I.A., Al-Sheikh H., Tasleem J.A., Haq Q.M.R. (2020). Plant-based phytochemicals as possible alternative to antibiotics in combating bacterial drug resistance. Antibiotics.

[82] Bouarab-Chibane L., Forquet V., Lantéri P., Clément Y., Léonard-Akkari L., Oulahal N., Degraeve P., Bordes C. (2019). Antibacterial properties of polyphenols: Characterization and QSAR (Quantitative Structure-Activity Relationship) models. Front. Microbiol..

[83] Puupponen-Pimiä R., Nohynek L., Meier C., Kähkönen M., Heinonen M., Hopia A., Oksman-Caldentey K.M. (2001). Antimicrobial properties of phenolic compounds from berries. J. Appl. Microbiol..

[84] Langeveld W.T., Veldhuizen E.J., Burt S.A. (2014). Synergy between essential oil components and antibiotics: A review. Crit. Rev. Microbiol..

[85] Lambert R.J., Skandamis P.N., Coote P.J., Nychas G.J. (2001). A study of the minimum inhibitory concentration and mode of action of oregano essential oil, thymol and carvacrol. J. Appl. Microbiol..

[86] Roy R., Tiwari M., Donelli G., Tiwari V. (2018). Strategies for combating bacterial biofilms: A focus on anti-biofilm agents and their mechanisms of action. Virulence.

[87] Slobodníková L., Fialová S., Rendeková K., Kováč J., Mučaji P. (2016). Antibiofilm activity of plant polyphenols. Molecules.

[88] Farhadi F., Khameneh B., Iranshahi M., Iranshahy M. (2019). Antibacterial activity of flavonoids and their structure-activity relationship: An update review. Phytother Res..

[89] Papuc C., Goran G.V., Predescu C.N., Nicorescu V., Stefan G. (2017). Plant polyphenols as antioxidant and antibacterial agents for shelf-life extension of meat and meat Products: Classification, structures, sources, and action mechanisms. Compr. Rev. Food Sci. Food Saf..

[90] Xie Y., Yang W., Tang F., Chen X., Ren L. (2015). Antibacterial activities of flavonoids: Structure-activity relationship and mechanism. Curr. Med. Chem..

[91] Sánchez-Maldonado A.F., Schieber A., Gänzle M.G. (2011). Structure-function relationships of the antibacterial activity of phenolic acids and their metabolism by lactic acid bacteria. J. Appl. Microbiol..

[92] Cueva C., Moreno-Arribas M.V., Martín-Alvarez P.J., Bills G., Vicente M.F., Basilio A., Rivas C.L., Requena T., Rodríguez J.M., Bartolomé B. (2010). Antimicrobial activity of phenolic acids against commensal, probiotic and pathogenic bacteria. Res. Microbiol..

[93] Tesaki S., Tanabe S., Moriyama M., Fukushi E., Kawabata J., Watanabe M. (1999). Isolation and identification of an antibacterial compound from grape and its application to foods. Health Environ. Res. Online.

[94] Baydar N.G., Göktürk G., Sağdiç O. (2004). Total phenolic contents and antibacterial activities of grape (*Vitis Vinifera* L.) extracts. Food Control.

[95] Baydar N.G., Sagdic O., Ozkan G., Cetin S. (2006). Determination of antibacterial effects and total phenolic contents of grape (*Vitis vinifera* L.) seed extracts. Int. J. Food Sci. Technol..

[96] Cheng V.J., El-Din A., Bekhit A., McConnell M., Mros S., Zhao J. (2012). Effect of extraction solvent, waste fraction and grape variety on the antimicrobial and antioxidant activities of extracts from wine residue from cool climate. Food Chem..

[97] Jayaprakasha G.K., Selvi T., Sakariah K.K. (2003). Antibacterial and antioxidant activities of grape (*Vitis vinifera*) seed extracts. Food Res. Int..

[98] Kačániová M., Terentjeva M., Kántor A., Felsöciová S., Puchalski C., Kunová S., Lopašovský L., Žiarovská J. (2018). Antimicrobial activity of *Vitis vinifera* L. pomace extract. Anim. Sci. Biotechnol..

[99] Silva V., Igrejas G., Falco V., Santos T.P., Torres C., Oliveira A.M.P., Pereira J.E., Amaral J.S., Poeta P. (2018). Chemical composition, antioxidant and antimicrobial activity of phenolic compounds extracted from wine industry by-products. Food Control.

[100] Xu C., Yagiz Y., Hsu W.Y., Simonne A., Lu J., Marshall M.R. (2014). Antioxidant, antibacterial, and antibiofilm properties of polyphenols from muscadine grape (*Vitis rotundifolia* Michx.) pomace against selected foodborne pathogens. J. Agric. Food Chem..

[101] Xu Y., Burton S., Kim C., Sismour E. (2015). Phenolic compounds, antioxidant, and antibacterial properties of pomace extracts from four Virginia-grown grape varieties. Food Sci. Nutr..

[102] Katalinić V., Možina S.M., Skroza D., Generalić I., Abramovič H., Miloš M., Ljubenkov I., Piskernik S., Pezo I., Terpinc P. (2010). Polyphenolic profile, antioxidant properties and antimicrobial activity of grape skin extracts of 14 *Vitis vinifera* varieties grown in Dalmatia (Croatia). Food Chem..

[103] Serra A.T., Matias A.A., Nunes A.V.M., Leitão M.C., Brito D., Bronze R., Silva S., Pires A., Crespo M.T., San Romão M.V. (2008). In vitro evaluation of olive- and grape-based natural extracts as potential preservatives for food. Innov. Food Sci. Emerg. Technol..

[104] Ali N., Afrasiab H., Anwar S. (2019). Antibacterial activity of leaf extracts of seven grape cultivars against six strains of bacteria. Adv. Life Sci..

[105] Oliveira D.A., Salvador A.A., Smânia A., Smânia E.F., Maraschin M., Ferreira S.R. (2013). Antimicrobial activity and composition profile of grape (*Vitis vinifera*) pomace extracts obtained by supercritical fluids. J. Biotechnol..

[106] Özkan G., Sagdiç O., Göktürk Baydar N., Kurumahmutoglu Z. (2004). Antibacterial activities and total phenolic contents of grape pomace extracts. J. Sci. Food Agric..

[107] Gómez-Mejía E., Roriz C.L., Heleno S.A., Calhelha R., Dias M.I., Pinela J., Rosales-Conrado N., León-González M.E., Ferreira I.C.F.R., Barros L. (2021). Valorisation of black mulberry and grape seeds: Chemical characterization and bioactive potential. Food Chem..

[108] Felhi S., Baccouch N., Ben Salah H., Smaoui S., Allouche N., Gharsallah N., Kadri A. (2016). Nutritional constituents, phytochemical profiles, in vitro antioxidant and antimicrobial properties, and gas chromatography-mass spectrometry analysis of various solvent extracts from grape seeds (*Vitis vinifera* L.). Food Sci. Biotechnol..

[109] Radulescu C., Buruleanu L.C., Nicolescu C.M., Olteanu R.L., Bumbac M., Holban G.C., Simal-Gandara J. (2020). Phytochemical profiles, antioxidant and antibacterial activities of grape (*Vitis vinifera* L.) seeds and skin from organic and conventional vineyards. Plants.

[110] Stefanović O.D. (2018). Synergistic Activity of Antibiotics and Bioactive Plant Extracts: A Study against Gram-Positive and Gram-Negative Bacteria. Bacterial Pathogenesis and Antibacterial Control.

[111] Kawabata K., Yoshioka Y., Terao J. (2019). Role of intestinal microbiota in the bioavailability and physiological functions of dietary polyphenols. Molecules.

[112] Costa J.R., Xavier M., Amado I.R., Gonçalves C., Castro P.M., Tonon R.V., Cabral L.M.C., Pastrana L., Pintado M.E. (2021). Polymeric nanoparticles as oral delivery systems for a grape pomace extract towards the improvement of biological activities. Mater. Sci. Eng. C Mater. Biol. Appl..

[113] Aiyegoro O.A., Okoh A.I. (2009). Use of bioactive plant products in combination with standard antibiotics: Implications in antimicrobial chemotherapy. J. Med. Plants Res..

[114] Siriwong S., Teethaisong Y., Thumanu K., Dunkhunthod B., Eumkeb G. (2016). The synergy and mode of action of quercetin plus amoxicillin against amoxicillin-resistant *Staphylococcus epidermidis*. BMC Pharm. Toxicol..

[115] Sanhueza L., Melo R., Montero R., Maisey K., Mendoza L., Wilkens M. (2017). Synergistic interactions between phenolic compounds identified in grape pomace extract with antibiotics of different classes against *Staphylococcus aureus* and *Escherichia coli*. PLoS ONE.

[116] Cushnie T.P., Lamb A.J. (2011). Recent advances in understanding the antibacterial properties of flavonoids. Int. J. Antimicrob. Agents.

